# Circ_0070934 promotes MGAT3 expression and inhibits epithelial-mesenchymal transition in bronchial epithelial cells by sponging miR-199a-5p

**DOI:** 10.1186/s13223-024-00890-y

**Published:** 2024-03-23

**Authors:** Ziqi Ding, Xinru Xiao, Liang Fan, Zhengdao Mao, Chuang Sun, Na Li, Qian Zhang

**Affiliations:** 1 Department of Respiratory and Critical Care Medicine, The Affiliated Changzhou Second People’s Hospital of Nanjing Medical University, Changzhou, 213164 China; 2 Changzhou Medical Center, Nanjing Medical University, Changzhou, 213164 China

**Keywords:** Asthma, Epithelial-mesenchymal transition, circ_0070934, miR-199a-5p, Mannoside acetylglucosaminyltransferase 3

## Abstract

**Background:**

Circular RNA (circRNA) has the potential to serve as a crucial regulator in the progression of bronchial asthma. The objective of this investigation was to elucidate the functional dynamics of the circ_0070934/miR-199a-5p/Mannoside acetylglucosaminyltransferase 3 (MGAT3) axis in the development of asthma.

**Methods:**

Circ_0070934, miR-199a-5p and MGAT3 in peripheral venous blood of 38 asthmatic patients and 43 healthy controls were detected by qRT-PCR, and the expression of MGAT3 protein was examined by ELISA. The GSE148000 dataset was analyzed for differences in MGAT3. The BEAS-2B cells were transfected with circ_0070934 plasmid and small interfering RNA, miR-199a-5p mimics and inhibitors. The apoptosis level was detected by flow cytometry and MGAT3 was detected by qRT-PCR and Western blot. The expression of E-cadherin, N-cadherin, Vimentin was examined by Western blot. Interleukin-4 (IL-4) and IL-13 were used to co-stimulate BEAS-2B cells as an asthmatic airway epithelial cell model. BEAS-2B cells exposed to type 2 cytokines (IL-4 and IL-13) were treated with circ_0070934 plasmid, and the expression of E-cadherin, N-cadherin, and Vimentin was detected by Western blot. The binding relationships were verified using dual-luciferase reporter assay and miRNA pull-down assay.

**Results:**

The expression of circ_0070934 and MGAT3 in peripheral venous blood of asthmatic patients was down-regulated, and the expression of miR-199a-5p was up-regulated. And the expression of MGAT3 was reduced in sputum of asthma patients. Down-regulating the expression of circ_0070934 could promote apoptosis of BEAS-2B cells and increase epithelial-mesenchymal transition (EMT), and this effect can be partially reversed by down-regulating miR-199a-5p. Circ_0070934 could inhibit the process of epithelial mesenchymal transition induced by IL-4 and IL-13 in BEAS-2B cells. In addition, miR-199a-5p could respectively bind to circ_0070934 and MGAT3.

**Conclusion:**

The findings of this study indicate that circ_0070934 may function as a competitive endogenous RNA (ceRNA) of miR-199a-5p, thereby modulating the expression of MGAT3 and impacting the process of EMT in bronchial epithelial cells. These results contribute to the establishment of a theoretical framework for advancing the prevention and treatment strategies for asthma.

## Introduction

Bronchial asthma is a recurrent chronic airway inflammatory disease characterized by chronic airway inflammation, airway hyperresponsiveness, reversible airflow restriction, and airway remodeling [1]. Among them, airway remodeling is the pathological basis of abnormal lung function and the root cause of asthma that is difficult to treat. The mechanisms of airway remodeling in asthma are currently well explored, among which epithelial-mesenchymal transition (EMT) is considered to be the key mechanism. EMT can lead to the fibrosis of asthmatic airway epithelium and promote smooth muscle hyperplasia, which disrupts the epithelial barrier, aggravates airway inflammation, and plays an important role in airway remodeling [2]. IL-4 and IL-13, as key type-2 inflammatory cytokines, could disrupt the barrier function of epithelial cells, leading to accumulation of cellular matrix proteins and EMT progress, and consequently participate in airway remodeling in asthma [3–5]. Therefore, the search for therapeutic targets related to the EMT process in asthma is of great interest.

CircRNA is a special class of non-coding RNA molecules, which is not easily degraded by exonucleotide enzymes and exists stably in cells [6]. Many studies have shown that circRNA is involved in the pathogenesis of asthma, such as regulating immune response, bronchial epithelial inflammation and airway remodeling [7–9]. In previous studies, circ_0070934 has been found to affect the occurrence and development of skin cancer [10], but whether it is involved in the pathophysiological process of asthma remains to be further studied. Our previous research found low expression of circ_0070934 in asthmatics by high-throughput sequencing, and predicted the binding of circ_0070934 to miR-199a-5p by circbank.

MiR-199a-5p is a miRNA of 22 nucleotides in length. It participates in a variety of cellular biological processes, including cell proliferation, differentiation, apoptosis, immune response, etc. It has been reported that the expression of miR-199a-5p is increased in peripheral blood and induced sputum of patients with neutrophilic asthma, and is negatively correlated with lung function [11]. Another study has shown that miR-199a-5p could regulate mesenchymal stem cell senescence in patients with idiopathic pulmonary fibrosis by regulating Sirt1/AMPK signaling pathway [12]. Previous studies have shown that certain circRNAs can bind competitively to miR-199a-5p to form miR-199a-5p response elements, thereby reducing their negative regulation of target genes, that is, influencing occurrence and development of non-small cell lung cancer through the competitive endogenous RNA (ceRNA) mechanism [13, 14]. However, whether it is involved in the biological process of asthma through the ceRNA mechanism, affecting bronchial epithelial cells apoptosis and EMT, remains unclear.

We preliminarily predicted the targeted regulation of mannoside acetylglucosaminyltransferase 3 (MGAT3) by miR-199a-5p using Targetscan, and found that the binding site was the same as that of circ_0070934. Hence, the present study aimed to confirm that circ_0070934 could reverse the EMT process of bronchial epithelial cells induced by IL-4 and IL-13. Mechanistically, circ_0070934 regulated the expression of MGAT3 gene by targeting miR-199a-5p as a ceRNA and affect the EMT process of bronchial epithelial cells, thus affecting the progression of asthma.

## Materials and methods

### Research objects and blood sample collection

Clinical peripheral venous blood samples were obtained from a total of 81 asthmatic patients and healthy controls at Changzhou Second People's Hospital during a period spanning from June 2020 to December 2021. The cohort consisted of two distinct groups: a healthy control group of 43 individuals, and an asthma group of 38 patients. The diagnostic criteria for asthma were in accordance with the 2020 edition of GINA [15]. The healthy control group was healthy volunteers with no history of asthma, allergic rhinitis, and other allergic and immune system disorders. The asthmatics were newly diagnosed and did not use anti-asthmatic drugs. All subjects should be excluded from comorbidities such as infection, pulmonary embolism, chronic obstructive pulmonary disease, pulmonary tuberculosis, blood system diseases and abnormal liver function. Blood routine, liver function, kidney function, blood glucose, blood lipids and electrocardiograms were performed to rule out underlying conditions. All subjects need to sign informed consent, and this study was approved by the Ethics Committee of Changzhou Second People's Hospital affiliated to Nanjing Medical University ([2020] KY213⁃01). We used EDTA anticoagulant tubes to collect peripheral vein of patients and healthy controls, and all participants' blood samples were stored in − 80 °C for the purpose of ascertaining circ_0070934, miR-199a-5p, and MGAT3 expression.

### Cell culture

Human lung epithelial cell line (BEAS-2B) was obtained from Zhongqiaoxinzhou Biotech (Shanghai, China) and cultured in DMEM (Gibco, Carlsbad, CA, USA) supplied with 10% fetal bovine serum at 37 °C containing 5% CO_2_. Human recombinant IL-4 and IL-13 were procured from PeproTech (Suzhou, China).

### Transfection

Vigorously active cells were introduced into 6-well plates. Subsequently, we employed Lipofectamine®3000 (Invitrogen, Carlsbad, CA, USA), introducing circ_0070934 overexpressed plasmids and its control group (vector), circ_0070934 small interference RNAs (si-circ_0070934) and its control group (si-NC), miR-199a-5p inhibitors and its control group (inhibitor NC) as well as miR-199a-5p mimics and its control group (miR-NC) into the cells. All the genes were purchased from Suzhou Gemma gene. After six hours, we changed to complete medium with or without IL-4 and IL-13(10 ng/mL). Subsequent experiments were performed after 48 h. The specific gene sequences were shown in Table 1.

**Table 1:** Gene sequence

Gene	Sense (5ʹ-3ʹ)	Antisense (5ʹ-3ʹ)
hsa_circ_0070934	AATTCGGTTCCCTCAAAATTATAAAGGCAGGAAA GCTCAAGACAAAGAAATCCAACAAGGCTAGTGAT TTCAGTGATATGGAGAATTGGCCAACACCAAGTG AATTAGTGAACACTGGATTTCAGAGCGTCCTCAG CCAAGGAAATAAAAAGCCACAAAATAGAAAAGAA AAAGAAGAGAAGGTTGAAAAGAGAAGTAACAGTG ACAGCAAAGAAAACCGGGAAACAAAATTAAATGG TCCTGGTGAAAACGTCAGTGAGGATGAGGCTCAG TCAAGTAATCAACGTAAGAGAGCTAATAAGCACA AGTGGGTACCACTCCACTTAGATGTTGTAAGATC AGAGAGTCAAGAAAGACCTGGATCCCGGAACAGC TCAAGATGTCAACCTGAAGCAAATAAACCAACAC ATAACAATAGGAGAAATGATACACGAAGTTGGAA GCGAGATAGAGAAAAAAGGGATGATCAAGATGAC GTTTCCAGTGTGAGAAGTGAGGGTGGTAATATCC GAGGTTCCTTTAGAGGTCGAGGAAGAGGCCGAGG ACGGGGAAGAGGACGAGGCAGAGGAAATCCTCGA TTGAACTTTGATTATTCATATGGTTATCAAGAAC ATGGTGAAAGGACTGATCAACCATTTCAAACAGA ACTTAATACCAGTATGATGTATTACTATGATGAT GGTACAGGTGTACAGGTGTATCCTGTGGAAGAAG CATTGCTTAAAGAGTATATTAAGCGTCAAAT	
si-circ_0070934	AAGCGUCAAAUAAUUCGGUTT	ACCGAAUUAUUUGACGCUUTT
negative control (si-NC)	UUCUCCGAACGUGUCACGUTT	ACGUGACACGUUCGGAGAATT
hsa-miR-199a-5p mimics	CCCAGUGUUCAGACUACCUGUUC	ACAGGUAGUCUGAACACUGGGUU
negative control (miR-NC)	UUCUCCGAACGUGUCACGUTT	ACGUGACACGUUCGGAGAATT
hsa-miR-199a-5p inhibitor	GAACAGGUAGUCUGAACACUGGG	
Inhibitor NC	CAGUACUUUUGUGUAGUACAA	
h-circ0070934-miR199a-5p-wt	ATGGAGAATTGGCCAACACCAAGTGAATTAGT **GAACACTGG**ATTTCAGAGCGTCCTCAGCCAAGGAAAT	
h-circ0070934-miR199a-5p-mut	ATGGAGAATTGGCCAACACCAAGTGAATTAGT **CTTGTGACC**ATTTCAGAGCGTCCTCAGCCAAGGAAAT	

### Real-time quantitative polymerase chain reaction (qRT-PCR)

Peripheral blood was utilized for total RNA extraction employing RNA liquid overspeed whole blood total RNA extraction kit (Hutian Oriental Technology, Beijing, China). For BEAS-2B cells, Cell/Tissue Total RNA Isolation Kit (Vazyme, Nanjing, China) was employed for extracting total RNA. The purity of RNA was measured using an ultraviolet spectrophotometer. First Strand cDNA Synthesis Kit and miRNA 1st Strand cDNA Synthesis Kit (Vazyme, Nanjing, China) were employed to perform reverse transcription of the extracted RNA into cDNA. Subsequently, qRT-PCR (ABI, Foster City, CA, USA) was carried out for the quantification of the RNA expression levels, utilizing AceQ qPCR SYBR Green Master Mix and miRNA Universal SYBR qPCR Master Mix (Vazyme, Nanjing, China). In peripheral blood tests, the internal parameters of circ_0070934, MGAT3, and mi-199a-5p were GAPDH, GAPDH + ACTB, and U6, respectively. In cell experiments, we regarded β-actin as a house-keeping gene for circ_0070934 and MGAT3, and regarded U6 as a house-keeping gene for miR-199a-5p. We assessed the result using the 2^− ΔΔCt^ method. The specific primers used for qRT-PCR were presented in Table 2.

**Table 2:** Primers used for qRT-PCR

Gene name		Sequence
circ_0070934	Forward	5′-ATCCTGTGGAAGAAGCATTGCTTAA-3′
	Reverse	5′-TCACTTGGTGTTGGCCAATTC-3′
MGAT3	Forward	5′-ATGAAGATGAGACGCTACAAGC-3′
	Reverse	5′-GCTGGACACCAGGTTAGGG-3′
β-actin	Forward	5′-CGTGGACATCCGCAAAGA-3′
	Reverse	5′-GAAGGTGGACAGCGAGGC-3′
GAPDH	Forward	5′-TCGACAGTCAGCCGCATCTTCTTT-3′
	Reverse	5′-ACCAAATCCGTTGACTCCGACCTT-3′
ACTB	Forward	5′-TCCGCAAAGACCTGTACGC-3′
	Reverse	5′-CTGGAAGGTGGACAGCGAG-3′
miR-199a-5p	Forward	5′-TAGCGGCCCAGTGTTCAGACTAC-3′
	Reverse	5′-AGTGCAGGGTCCGAGGTATT-3′
U6	Forward	5′-CGCTTCGGCAGCACATATAC-3′
	Reverse	5′-TTCACGAATTTGCGTGTCATC-3′

### Enzyme-linked immunosorbent assay (ELISA)

ELISA kit (Beyotime, Shanghai, China) was used to detect the level of MGAT3 protein in plasma samples of asthma patients and healthy controls, and the experiment and operation were conducted in strict accordance with the kit instructions.

### Western blot

BEAS-2B cells were lysed in RIPA lysis buffer supplied with protease and phosphatase inhibitor cocktail (Beyotime, Shanghai, China). Protein concentrations were determined using a BCA Assay kit from Beyotime. Protein specimens were electrophoresed on 10% sodium dodecyl sulfate polyacrylamide gel, and transferred onto polyvinylidene membrane. After blocking using phosphate buffered saline with tween-20 (PBST) blocking buffer (Beyotime, Shanghai, China) for 30 min, the blots were incubated with primary antibodies against E-cadherin, N-cadherin, Vimentin (Beyotime, Shanghai, China), MGAT3, and GAPDH (Abcam, Cambridge, UK) at 4 °C over night. Subsequently, the membranes underwent three consecutive washes using tris-buffered saline with tween-20 (TBST) as well as exposed to goat anti-rabbit IgG secondary antibody (Abcam, Cambridge, UK) at room temperature for 1.5 h. After washing, protein visualization and quantification were performed by enhanced chemiluminescence kit (Tanon, Shanghai, China). Protein evaluation was conducted through densitometric analysis through the ImageJ software (National Institutes of Health, Bethesda, MD, USA).

### Flow cytometry

Apoptosis of BEAS-2B cells were measured with Annexin V-FITC/PI Apoptosis Detection Kits (Vazyme, Nanjing, China) following manufacturer’s instructions. We collected the treated BEAS-2B from 6-well plates with trypsin, washed twice with pre-cooled phosphate saline buffer (PBS), subsequent to that, centrifugation was carried out at 1800 rpm for a duration of 5 min. Then we re-suspended them with 100 mL binding buffer, and added 5 μl PI staining solution and 5 μl Annexin V-FITC. Cells were kept in the dark for a 10 min incubation period. Finally, we added a 400 mL binding buffer prior to the analysis. Subsequently, the cells were analyzed utilizing flow cytometer (Becton Dickinson and Co, Franklin Lakes, NJ, USA). The apoptosis rates were determined by analyzing the percentage of cells that had either died or were undergoing apoptosis.

### Dual-luciferase reporter assay

Circ_0070934 and MGAT3 wild-type (WT) 3'UTR sequences, along with circ_0070934 and MGAT3 mutant (MUT) 3'UTR sequences, were generated through Suzhou Gemma gene. Subsequently, 5 × 10^5^ BEAS-2B cells seedings got performed in 24-well plates as well as transfected utilizing 500 ng WT or MUT plasmid, 50 nM miR-199a-5p mimics or negative control (NC), along with Lipofectamine®3000 (Thermo Fisher Scientific, Inc.) for 6 h at 37 °C. After 48 h of transfection, cells were harvested under 4 °C for 20 min using lysate from Dual-Luciferase®Reporter Assay System (Promega Corp.), then placed at − 80 °C refrigerator overnight. Finally, we detected firefly luminescence and renilla luminescence with the enzyme labeling instrument.

### MiRNA pull-down

Biotin-labeled miR-199a-5p probes (probe sequences: CCCAGUGUUCAGACUACCUGUUC) and biotin-labeled NC negative control probes (probe sequences: UCACAACCUCCUA GAAAGAGUAGA) were synthesized by BersinBio (Guangzhou, China) and transfected into BEAS-2B cells. The concentration of the transfection probe was 100 nM for 48 h of transfection. We used the miRNA pulldown kit (Bes5108, BersinBio) to perform the miRNA pull-down assay. The miRNA-RNA complex in the cell lysate was pulled off with streptavidin magnetic beads, and the complex bounded to the magnetic beads was eluted with washing buffer. The enrichment of circ_0070934 pulled by the miR-199a-5p probe was detected by qRT-PCR.

### Bioinformatics analysis

The datasets were screened from the GEO database (http://www.ncbi.nlm.nih.gov/geo) with the following selection criteria: 1. The dataset must contain genome-wide expression mRNA microarray data; 2. The dataset should include sputum samples from asthmatic patients and healthy controls. Based on the criteria the gene expression profiling dataset GSE148000 was obtained. GSE148000 contained 9 sputum samples from asthmatic patients and 7 sputum samples from healthy controls. The expression of MGAT3 in the asthma and control groups was obtained using the R package Limma.

### Statistical analysis

GraphPad Prism v.8.0 (GraphPad Software Inc., San Diego, CA, USA) was used for the data analysis. To assess the differences between groups, pairwise comparisons between two groups were conducted using T-test, and for comparing multiple cohorts, one-way analysis of variance (ANOVA) was employed, as appropriate. The results were presented as mean ± standard error (SEM) based on data gathered from three independent experiments. Statistical significance was determined at a significance level of *P* Value < 0.05.

## Results

### Expressions of circ_0070934, miR-199a-5p and MGAT3 were different in peripheral blood of asthmatic patients and healthy controls

**Fig. 1:**
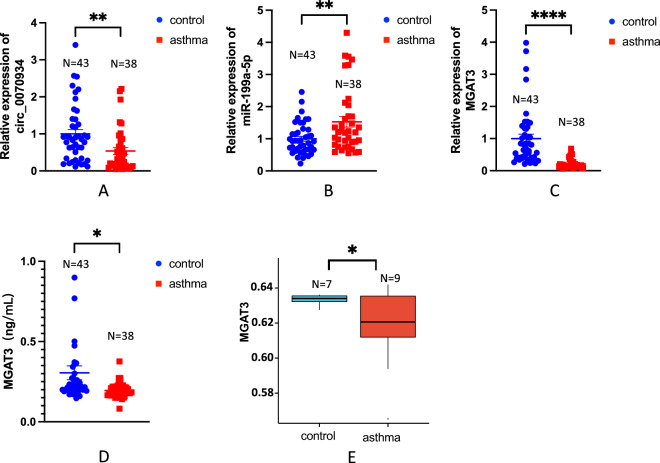
circ_0070934, miR-199a-5p and MGAT3 were differentially expressed in asthma patients and healthy controls. **A–C** The expression of circ_0070934, miR-199a-5p and MGAT3 in peripheral venous blood of healthy controls (N = 43) and asthmatic patients (N = 38) was determined by qRT-PCR. **D** ELISA was used to detect the expression level of MGAT3 protein in peripheral plasma of healthy controls (N = 43) and asthmatic patients (N = 38). **E** The expression level of MGAT3 in sputum of healthy controls (N = 7) and asthmatic patients (N = 9) was presented by analyzing the GSE148000 gene expression profiling dataset. The data were presented as mean ± SEM. qRT-PCR and ELISA assays were repeated three times. ns, no significant, *P* > 0.05; *, *P* < 0.05; **, *P* < 0.01; ***, *P* < 0.001; ****, *P* < 0.0001

The study included 38 asthmatic patients and 43 healthy controls. Age, sex, and body mass index (BMI) got standardized between these two cohorts, as detailed in Table 3. The qRT-PCR results revealed notable differences. Specifically, compared to the healthy controls, the expression of circ_0070934 and MGAT3 was significantly decreased (Fig. 1A and C), while the expression of miR-199a-5p was significantly increased (Fig. 1B) in asthmatic patients. Meanwhile, ELISA results showed that the protein expression of MGAT3 in peripheral blood of asthmatic patients was significantly decreased compared with that of the healthy controls (Fig. 1D). In addition, we found that the expression of MGAT3 in sputum of asthmatic patients was lower than healthy controls by bioinformatics analysis (Fig. 1E).

**Table 3:** Clinical characteristics of the subjects

Groups	Control (N = 43)	Asthma (N = 38)	*P*
Sex (male/female)	27/16	22/16	0.653
Age (y)	40.30 ± 10.40	40.73 ± 13.65	0.738
BMI (kg/m^2^)	26.43 ± 3.51	25.30 ± 3.46	0.606
FEV_1_% pre	108 ± 8.67	88.19 ± 27.27	0.024
FEV_1_/FVC (%)	83.00	73.55	0.016
	(97.70–114.10)	(67.13–82.00)	
EOS (× 10^9^/L)	0.12 (0.07–0.21)	0.36 (0.20–0.71)	< 0.001
EOS%	1.80 ± 1.33	6.00 ± 4.38	< 0.001
FeNO (ppb)	19.00	61.00	< 0.001
	(14.00–27.00)	(26.50–93.50)	

*BMI* body mass index, *FEV*_*1*_*% pre* percent of predicted forced expiratory volume in 1 s, *FVC* forced vital capacity, *EOS* eosinophils, *FeNO* fraction of exhaled nitric oxide

### Regulation of circ_0070934 and miR-199a-5p affected the expression of MGAT3

**Fig. 2:**
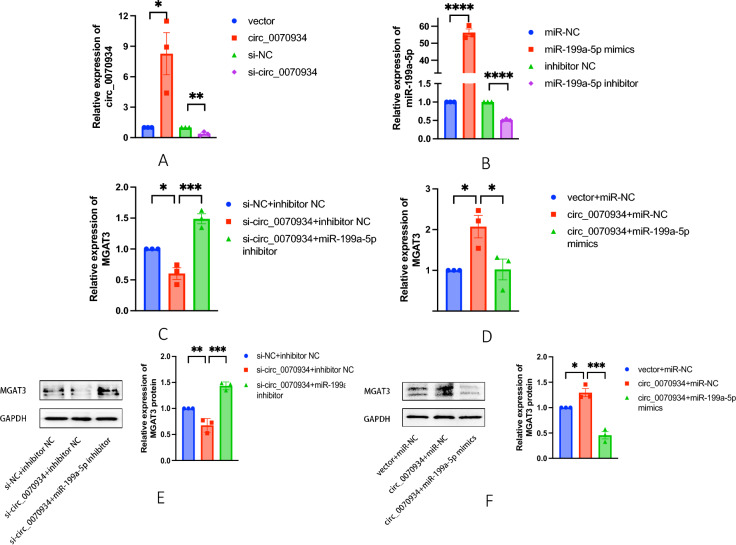
Regulation of the expression of circ_0070934 and miR-199a-5p could affect the expression of MGAT3. **A** qRT-PCR was used to detect the expression of circ_0070934 after transfection of vector or circ_0070934 plasmid, and si-NC or si-circ_0070934 in BEAS-2B cells. **B** The expressions of miR-199a-5p in BEAS-2B cells after overexpression and knockdown miR-199a-5p was measured by qRT-PCR. **C** and **D** mRNA levels of MGAT3 in BEAS-2B cells after regulating circ_0070934 and miR-199a-5p was detected by qRT-PCR. **E** and **F** The expression level of MGAT3 protein in BEAS-2B cells was determined by Western blot. The data were presented as mean ± SEM. Our experiments were repeated three times. ns, no significant; *P* > 0.05; *P < 0.05; **P < 0.01; ***P < 0.001; ****P < 0.0001

To comprehend the interplay between circ_0070934, miR-199a-5p, and MGAT3 in bronchial epithelial cells, we performed a transfection of vector, circ_0070934 plasmid and siRNA into BEAS-2B cells. Successful transfections were verified using qRT-PCR (Fig. 2A). Subsequently, we transfected BEAS-2B cells with miR-199a-5p mimics or negative control (miR-NC), as well as miR-199a-5p inhibitor or negative control (inhibitor NC), and confirmed the transfection efficacy by qRT-PCR (Fig. 2B). qRT-PCR demonstrated that down-regulation of circ_0070934 significantly reduced MGAT3 expression in BEAS-2B cells (Fig. 2C). Interestingly, the combined down-regulation of circ_0070934 and miR-199a-5p effectively reversed the down-regulation of MGAT3 expression (Fig. 2C). Meanwhile, elevated levels of circ_0070934 resulted in a significant upregulation of MGAT3 expression, and co-overexpression of circ_0070934 and miR-199a-5p successfully counteracted the up-regulation of MGAT3 expression (Fig. 2D). This pattern was further highlighted by the corresponding alterations in the MGAT3 protein levels in BEAS-2B cells, as illustrated in Fig. 2E and F.

### Circ_0070934 affected apoptosis and EMT of BEAS-2B cells

**Fig. 3:**
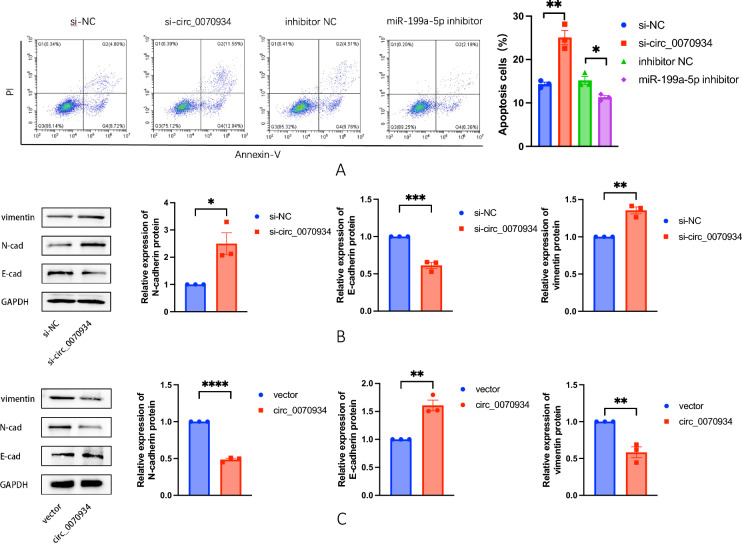
Down-regulation of circ_0070934 can promote apoptosis and affect the process of EMT in BEAS-2B cells. **A** BEAS-2B cells were divided into four groups: si-NC, si-circ_0070934, inhibitor NC and miR-199a-5p inhibitor. The level of cell apoptosis after transfection was quantified by Flow cytometry. **B** and **C** Western blot was used to detect the expression levels of epithelial-mesenchymal transition related molecular markers, including E-cadherin, N-cadherin and vimentin after transfection of si-NC or si-circ_0070934, and vector or circ_0070934 plasmid in BEAS-2B cells. The data were presented as mean ± SEM. Our experiments were repeated three times. ns, no significant; *P* > 0.05; *P < 0.05; **P < 0.01; ***P < 0.001; ****P < 0.0001

Apoptosis in BEAS-2B cells was assessed by flow cytometry, and EMT-related marker proteins were quantified using Western blot analysis. Flow cytometry results showed that apoptosis of BEAS-2B cells increased after circ_0070934 silencing, while apoptosis of BEAS-2B cells decreased after miR-199a-5p silencing (Fig. 3A). In terms of Western blot results, circ_0070934 knockdown resulted in decreased E-cadherin expression and increased N-cadherin and vimentin expressions in BEAS-2B cells (Fig. 3B). Conversely, overexpressed circ_0070934 resulted in opposite outcomes (Fig. 3C). These results suggested that the down-regulation of circ_0070934 expression can promote the process of apoptosis and EMT of bronchial epithelial cells.

### Circ_0070934 regulated apoptosis and EMT through miR-199a-5p

**Fig. 4:**
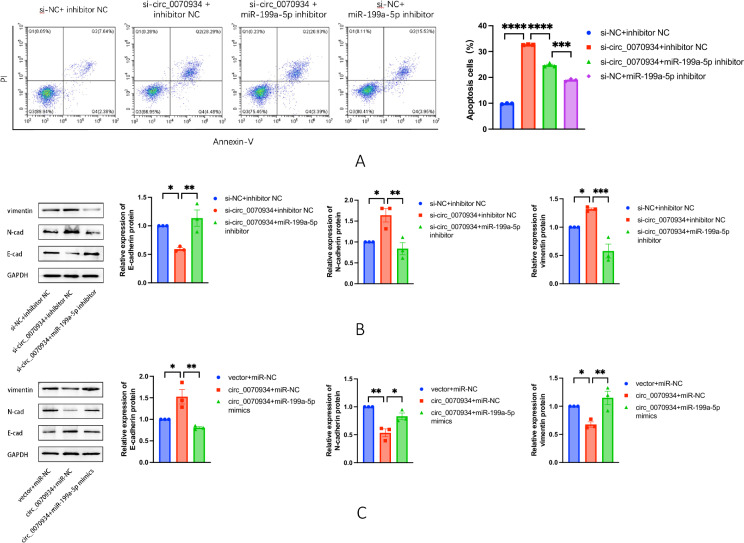
MiR-199a-5p could reverse the effect of circ_0070934 on apoptosis and EMT of BEAS-2B cells. **A** BEAS-2B cells were divided into four groups: si-NC + inhibitor NC, si-circ_0070934 + inhibitor NC, si-circ_0070934 + miR-199a-5p inhibitor, si-NC + miR-199a-5p inhibitor. Flow cytometry was used to measure the level of cell apoptosis. **B** BEAS-2B cells were divided into three groups: si-NC + inhibitor NC, si-circ_0070934 + inhibitor NC, and si-circ_0070934 + miR-199a-5p inhibitor. The expression levels of proteins associated with EMT was detected by Western blot. **C** BEAS-2B cells were divided into three groups: vector + miR-NC, circ_0070934 + miR-NC, and circ_0070934 + miR-199a-5p mimics. Western blot was used to determine the expression levels of proteins associated with EMT. The data were presented as mean ± SEM. Our experiments were repeated three times. ns, no significant; *P* > 0.05; *P < 0.05; **P < 0.01; ***P < 0.001; ****P < 0.0001

We conducted co-transfections of circ_0070934 siRNA and miR-199a-5p inhibitor into BEAS-2B. In comparison to circ_0070934 siRNA co-transfection with inhibitor NC, co-transfection circ_0070934 siRNA with miR-199a-5p inhibitor exhibited reduced apoptosis in BEAS-2B cells (Fig. 4A), along with an elevation in E-cadherin expression and a reduction in N-cadherin and vimentin expression (Fig. 4B). And compared with the co-transfected miR-199a-5p inhibitor and circ_0070934 siRNA group, the apoptosis of the cells in the co-transfected miR-199a-5p inhibitor and si-NC group was reduced more (Fig. 4A). Furthermore, we co-transfected BEAS-2B cells with circ_0070934 overexpressed plasmid and miR-199a-5p mimics. In comparison to the cells co-transfected with circ_0070934 overexpression plasmid and miR-NC, the cells co-transfected with circ_0070934 overexpression plasmid as well as miR-199a-5p mimics showed a significant down-regulation in E-cadherin expression, accompanied by a notable up-regulation in N-cadherin and vimentin expression (Fig. 4C). These outcomes indicated that the regulation of apoptosis and EMT by circ_0070934 in bronchial epithelial cells might be through miR-199a-5p.

### Circ_0070934 inhibited EMT process caused by type 2 cytokine-exposed BEAS-2B cells.

**Fig. 5:**
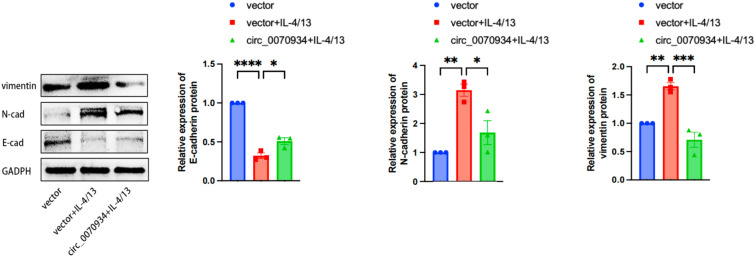
Circ_0070934 suppressed the EMT process induced by BEAS-2B cells exposed to IL-4 and IL-13. BEAS-2B cells were divided into three groups: vector, vector + IL4/13, and circ_0070934 + IL-4/13. Western blot was used to detect expression levels of proteins associated with EMT in BEAS-2B cells. The data were presented as mean ± SEM. Our experiments were repeated three times. ns, no significant; *P* > 0.05; *P < 0.05; **P < 0.01; ***P < 0.001; ****P < 0.0001

We added circ_0070934 plasmid or vector to BEAS-2B cells for 6 h, and then exchanged the medium with complete medium added with 10 ng/mL of IL-4 and IL-13. After 48 h of action we extracted proteins for Western Blot. The outcomes revealed increased expression of N- cadherin and vimentin and decreased expression of E-cadherin in type 2 cytokine-exposed BEAS-2B cells. While circ_0070934 could partially reversed this alteration (Fig. 5). This implied that circ_0070934 could suppress the EMT process in asthmatic airway epithelial cells.

### Circ_0070934 directly interacted with miR-199a-5p

**Fig. 6:**
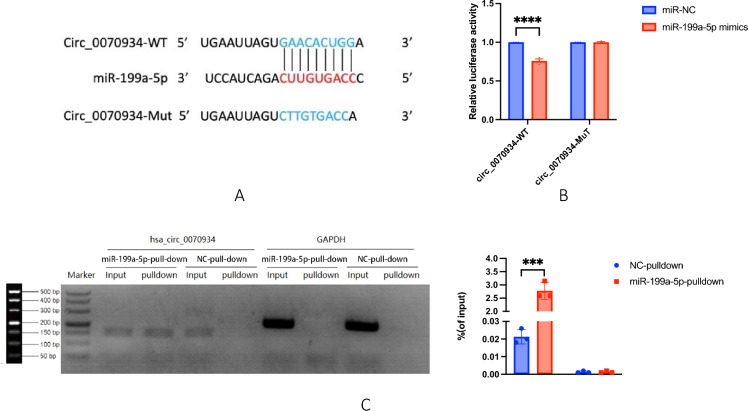
Circ_0070934 had a targeted binding relationship with miR-199a-5p. **A** There are potential base binding sites between circ_0070934 and miR-199a-5p. **B** Dual-luciferase reporter assay was conducted to explicit the binding relationship between circ_0070934 and miR-199a-5p. **C** MiR-199a-5p-pull-down and NC-pull-down were the two groups of cells transfected with miR-199a-5p and NC probes, respectively. The input was the sample control, equivalent to total RNA, and the pulldown was the RNA after magnetic bead pull-down. There are four groups of RNA. The expression of hsa circ 0070934 and GAPDH were detected by qPCR. The data were presented as mean ± SEM. Our experiments were repeated three times. ns, no significant; *P* > 0.05; *P < 0.05; **P < 0.01; ***P < 0.001; ****P < 0.0001

The prediction of the circbank gene suggested a potential binding between circ_0070934 and miR-199a-5p. In order to validate this interaction, we performed a dual-luciferase reporter gene assay. Co-transfection of BEAS-2B cells was conducted using either circ_0070934-WT or circ_0070934-Mut plasmids, along with miR-199a-5p mimics or negative controls (Fig. 6A**)**. The findings conclusively indicated that upon introduction of miR-199a-5p mimics, there was a significant reduction in luciferase activity in the circ_0070934-WT reporter gene. Conversely, there was no notable impact on the luciferase activity in the circ_0070934-Mut reporter gene (Fig. 6B). The experimental evidence established the specific binding interactions between miR-199a-5p and circ_0070934. Furthermore, the outcomes of miRNA pull-down assay exhibited enriching circ_0070934 in biotin-labeled miR-199a-5p (Fig. 6C), confirming the interaction of miR-199a-5p and circ_0070934.

### MGAT3 was a downstream target of miR-199a-5p

**Fig. 7:**
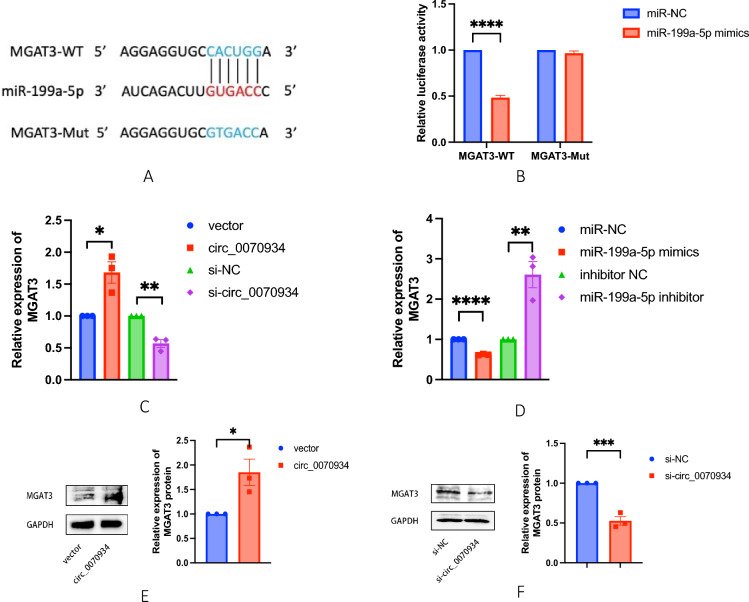
MGAT3 was a downstream target of miR-199a-5p. **A** There are potential base binding sites between MGAT3 and miR-199a-5p. **B** Dual-luciferase reporter assay was used to verify the binding relationship between MGAT3 and miR-199a-5p. **C** BEAS-2B cells were divided into four groups: vector, circ_0070934, si-NC and si-circ_0070934. **D** BEAS-2B cells were divided into four groups: miR-NC, miR-199a-5p mimics, inhibitor NC and miR-199a-5p inhibitor. **C** and **D** mRNA expression level of MGAT3 was determined by qRT-PCR. **E** and **F** The protein expression level of MGAT3 was determined by Western blot. The data were presented as mean ± SEM. Our experiments were repeated three times. ns, no significant; *P* > 0.05; *P < 0.05; **P < 0.01; ***P < 0.001; ****P < 0.0001

**Fig.8:**
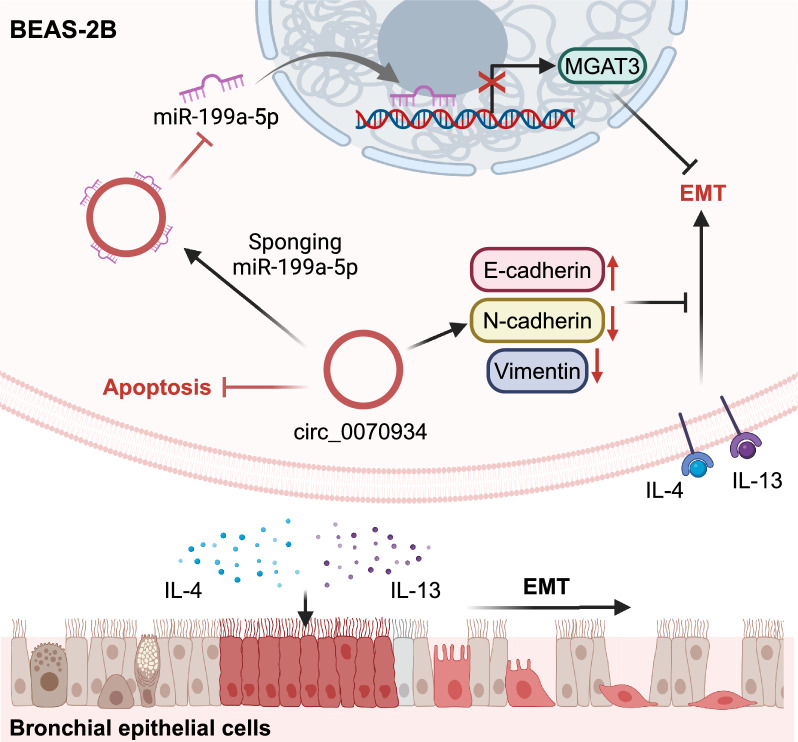
Schematic diagram demonstrating mechanisms by which circ_0070934 promotes MGAT3 expression and inhibits EMT progress in bronchial epithelial cells by sponging miR-199a-5p

Utilizing TargetScan, we embarked on a quest to identify potential miR-199a-5p targets, and found that the binding sites of MGAT3 and miR-199a-5p were highly consistent with those of circ_0070934 and miR-199a-5p. Seeking to examine the interaction between miR-199a-5p and MGAT3, we engineered plasmids harboring the MGAT3-WT and MGAT3-Mut sequences. These constructs were subsequently co-transfected with miR-199a-5p mimics or negative controls into BEAS-2B cells. MiR-199a-5p mimics profoundly curtailed the luciferase activity in the MGAT3-WT reporter gene, whereas the MGAT3-Mut reporter gene remained unaffected (Fig. 7A and B). This unequivocally indicated that miR-199a-5p could bind to MGAT3 and inhibit its transcription. Furthermore, our investigation extended to the quantification of MGAT3 expression via qRT-PCR and Western blot analysis. Notably, the overexpression of circ_0070934 led to an upregulation of MGAT3 in BEAS-2B cells (Fig. 7C and E), whereas circ_0070934 knockdown resulted in the downregulation of MGAT3 expression (Fig. 7C and F). Meanwhile, the overexpression of miR-199a-5p could lead to the downregulation of MGAT3 expression, and silencing of miR-199a-5p could lead to the upregulation of MGAT3 expression (Fig. 7D**)**. These collective findings substantiated the postulate that MGAT3 acted as a gene targeted by miR-199a-5p. It was negatively regulated by miR-199a-5p and positively regulated by circ_0070934 (Fig. 8).

## Discussion

CircRNA has been found to play an important regulatory role in asthma [16–18]. Nevertheless, the mechanism by which circRNA affects asthma remains unclear. At the same time, there is a lack of relevant clinical studies evaluating the correlation between circRNA and clinical data in asthmatic patients. In the present study, peripheral blood levels of circ_0070934, miR-199a-5p and MGAT3 were detected and shown to differ between asthmatic patients and healthy controls. Simultaneously, in vitro experiments further indicated that the downregulation of circ_0070934 could lead to increased EMT in bronchial epithelial cells, which may further affect airway remodeling and promote the progression of pulmonary fibrosis. And circ_0070934 suppressed the process of epithelial mesenchymal transition induced by IL-4 and IL-13 in BEAS-2B cells. Moreover, we confirmed that circ_0070934 can bind competitively to miR-199a-5p, resulting in increased expression of MGAT3. Therefore, circ_0070934, miR-199a-5p and MGAT3 were of some significance in the diagnosis of asthma and may be used as potential biomarkers for asthma diagnosis. Meanwhile, circ_0070934 can be used as a therapeutic target for asthma to inhibit the progression of airway remodeling and fibrosis.

Recent studies have shown that circRNA is involved in the occurrence and development of asthma, such as regulating the immune response, participating in bronchial epithelial inflammation and airway remodeling, which have important implications for the prevention, diagnosis and treatment of asthma. For example, a study has shown that circ_001372 inhibits the inflammatory response in asthmatic mice by regulating Sirt1/NFAT5 through miRNA-128-3p [17]. Another study suggested that the circ_0005519 expression level was high in asthmatics, and negatively correlated with the let-7a-5p expression. Circ_0005519 may increase IL-13/IL-6 level by adjusting let-7-a-5p to affect asthma [19]. A growing body of research is devoted to understanding the regulatory relationship between circRNA and asthma, and the mechanisms and biological functions of how circRNA influences the occurrence and development of asthma are being revealed.

EMT is a dynamic process in which epithelial cells undergo a remarkable transformation into a mesenchymal-like state. EMT is categorized into three distinct types, each characterized by its unique roles. Type I EMT primarily contributes to embryo implantation, organ development, and shaping of vital structures. Type II EMT, on the other hand, assumes a crucial role in the processes such as tissue repair, regenerative response, fibrotic remodeling, and orchestration of inflammatory reaction. In contrast, type III EMT is predominantly implicated in the progression and metastasis of cancerous growth. Of particular interest is type II EMT, which holds a special connection to atopic diseases. This variant exerts a profound influence on the rejuvenation of epithelial layers and the provocation of inflammatory cascades across various anatomical sites, including the lungs, nasal passages, intestines, and skin [20]. EMT is essential for normal tissue repair and signaling through multiple inflammatory pathways. Elevated EMT within bronchial epithelial cells can give rise to impaired barrier function in the context of bronchial asthma. The typical features of bronchial asthma are chronic airway inflammation, airway remodeling, airway hyperresponsiveness, and reversible airflow restriction. The airway remodeling process involves the increase of fibroblasts in the airway epithelium, eventually leading to fibrosis, and EMT may explain the origin of these fibroblasts [21]. Recently, it has been shown that house dust mite (HDM) extracts promote EMT processes in airway epithelial cells through stimulating CD146 and TGF-β/ MAD-3 signaling pathways by IL-33 [22].

So far, very little is known about circ_0070934. We only know that circ_0070934 is involved in the invasion and proliferation of skin squamous cell carcinoma [10, 23], but the specific mechanism is unclear. Meanwhile, miR-199a-5p has been extensively studied in tumor EMT. For instance, in ovarian cancer, breast cancer, larynx cancer, oral squamous cell cancer, thyroid cancer, colorectal cancer, invasive bladder cancer and other malignant tumors, miR-199a-5p had an inhibitory effect on EMT [24–29]. However, in other tumors, such as hepatocellular carcinoma, gastric cancer, cervical cancer and prostate cancer, the EMT process in tumor cells is promoted [30–33]. Despite this, the effects of circ_0070934 and miR-199a-5p on EMT process in bronchial epithelial cells remain unclear. In the present study, we found that circ_0070934 inhibited the EMT of airway epithelial cells, while miR-199a-5p had the opposite effect.

MGAT3 encodes N-acetylglucosamine transferase 3 (Gnt III), which is mainly derived from structural cells and is highly expressed in alveolar epithelial cells. It is also involved in the EMT process of various tumors and has a regulatory effect on the migration of tumor cells. In human hepatocellular carcinoma cells treated with TGF-β1 induction, reduced expression of the GnTIII gene and its catalytic product bisected GlcNAc structure, led to the up-regulation of Smad3 and Erk1/2 phosphorylation, which ultimately led to the EMT process of hepatocellular carcinoma [34]. Pinho et al. found that the loss of n-glycosylation of E-cadherin mediated by MGAT3 and GnT III was the mechanism of EMT, and the expression of MGAT3 was significantly reduced during EMT [35]. In addition, a recent study showed that in lung cancer, MGAT3 was regulated by miR-188-5p, inhibiting the EMT process and cancer cell metastasis [36]. In conclusion, an increasing number of studies have shown that MGAT3 could inhibit the EMT process in cancer cells, but its effect on EMT in airway epithelial cells remained understudied. Our investigation demonstrated that MGAT3 was negatively regulated by miR-199a-5p and positively regulated by circ_0070934, thus inhibiting the EMT process in bronchial epithelial cells. However, the specific mechanism of its effect needs to be further investigated.

In this study, we compared the circ_0070934, miR-199a-5p, and MGAT3 expressions in peripheral venous blood of both asthmatic patients and healthy controls and analyzed the expression of MGAT3 in the sputum of asthmatic patients and healthy controls in a database. In addition, we performed comprehensive experiments in human lung epithelial cells to confirm the role of circ_0070934 functioning as a ceRNA, regulating MGAT3 and subsequently inhibiting the EMT process in bronchial epithelial cells. Meanwhile, we further found that circ_0070934 could restrain EMT process in asthmatic airway epithelial cells. This provides new insights into the development of more effective treatment strategies for treating asthma. However, it should be pointed out that the number of patient samples in our clinical trial is limited, and the sample size needs to be expanded for further validation, and the collected clinical information of patients needs to be more comprehensive. Second, we lacked sufficient sputum or bronchoalveolar lavage fluid (BALF) samples to detect differences in the expression of circ_0070934, miR-199a-5p, and MGAT3 to further illustrate their relevance to asthma. Third, in the basic experiment, we selected only one cell line for the experiment, and there was a lack of in vivo experiments to confirm that circ_0070934 affected MGAT3 expression and EMT processes through the ceRNA mechanism. We will supplement the above data in future experiments.

## Conclusion

In summary, we found lower expression of circ_0070934 and MGAT3, and higher expression of miR-199a-5p in the peripheral blood of asthmatic patients compared to healthy controls. Through bioinformatics analysis, we found that the expression of MGAT3 in sputum of asthmatic patients was reduced compared with that of healthy controls. We confirmed that circ_0070934 can bind to miR-199a-5p to promote the expression of MGAT3 and inhibit EMT and apoptosis in BEAS-2B cells. This indicates that circ_0070934 can be used as a protective factor for asthma, which provides a new idea for asthma diagnosis and treatment. Nevertheless, we still need more studies to determine it.

## Data Availability

All data generated or analysed during this study are included in this published article [and its supplementary information files].

## Abbreviations

circRNA: Circular RNA
MGAT3: Mannoside acetylglucosaminyltransferase 3
IL: Interleukin
EMT: Epithelial-mesenchymal transition
ceRNA: Competitive endogenous RNA
qRT-PCR: Real-time quantitative polymerase chain reaction
ELISA: Enzyme-linked immunosorbent assay
PBS: Phosphate saline buffer
WT: Wild-type
MUT: Mutant
NC: Negative control
ANOVA: Analysis of variance
SEM: Mean ± standard error
BMI: Body mass index
HDM: House dust mite
Gnt III: N-acetylglucosamine transferase 3
BALF: Bronchoalveolar lavage fluid
